# Development of a Colloidal Gold Immunochromatographic Strip for Rapid Detection of Cyfra 21-1 in Lymph Node Metastasis of Thyroid Cancer

**DOI:** 10.3389/fbioe.2022.871285

**Published:** 2022-04-12

**Authors:** Lijie Xu, Shuhao Wang, Zhechen Wu, Chengcheng Xu, Xinwei Hu, Haitian Ding, Yanqiang Zhang, Bing Shen, Yehai Liu, Kaile Wu

**Affiliations:** ^1^ Department of Otorhinolaryngology, Head and Neck Surgery, The First Affiliated Hospital of Anhui Medical University, Hefei, China; ^2^ School of Basic Medicine, Anhui Medical University, Hefei, China; ^3^ The First Clinical Medical College, Anhui Medical University, Hefei, China

**Keywords:** thyroid cancer, CYFRA 21-1, immunochromatographic strip, rapid diagnosis technology, lymph node metastasis

## Abstract

Thyroid cancer is the most common endocrine tumor, and the rate of early lymph node metastasis may be as high as 60%. Currently, detection of lymph node metastasis of thyroid cancer during surgery is limited and time-consuming. Elevated levels of Cyfra 21-1, the proteolytic portion of cytokeratin, are associated with the metastasis and progression of thyroid cancer and are an effective biomarker for the prognosis and diagnosis of thyroid cancer. In this study, an immunochromatographic strip test based on colloidal gold nanoparticles was developed to semi-quantitatively detect the levels of Cyfra 21-1 in lymph nodes within 15 min. The standard (calibration) curve equation was Y = 0.003708 × X + 0.1101, and the detection limit was 0.55–1.14 ng mL^−1^. The strip did not detect other protein markers of epithelial cells at a concentration of 500 ng mL^−1^, including cytokeratin 8, cytokeratin 18, epithelial membrane antigen, and epidermal surface antigen. The ability of the strip to differentiate positive from negative metastasis in 40 lymph node specimens was 100% concordant with that of immunohistochemical staining for Cyfra 21-1. In an assessment of 20 lymph node specimens that had been determined by postoperative histopathology to be positive for lymph node metastasis and 20 specimens that were negative, the sensitivity and specificity of the strip were 100% and 95%, respectively. The sensitivity of the strip remained stable when stored at room temperature for 6 months. Together, these results indicated that although further testing using a larger sample size will be required, this immunochromatographic strip test may be useful for rapid intraoperative detection of thyroid cancer metastasis to lymph nodes.

## Introduction

In 2020, there were 586,202 newly diagnosed thyroid cancer cases worldwide, making it one of the most common endocrine tumors (Sung et al., 2021). Although many of these cases had a low degree of malignancy, approximately 40%–60% of patients with thyroid cancer experience early cervical lymph node metastasis (Smith et al., 2012; Tang et al., 2018). Detection of lymph node metastasis includes the use of rapid intraoperative frozen sections and postoperative histopathological examination. Obtaining results from frozen sections takes approximately 60 min during surgery, whereas obtaining postoperative pathological results typically takes 5–10 days. In addition, numerous studies have shown that the false negative rate of intraoperative histopathological examination is 5%–52% (Celebioglu et al., 2006; Zavagno et al., 2008; Horvath et al., 2009; Hoen et al., 2016). The results of frozen section and postoperative pathological examinations are dependent more on the subjective judgment of the pathologist than on objective findings (Roberts et al., 2003). If a rapid detection method can be developed to accurately determine intraoperative lymph node metastasis, the need for further lymph node dissection could be determined during surgery, which may reduce the opportunity for tumor metastasis and multiple operations. Therefore, rapid, accurate, and quantitative intraoperative detection of lymph node metastasis is of great importance to improve the quality of thyroid cancer surgery and the prognosis of patients with this cancer.

Tumor metastasis is a continuous and dynamic process involving the separation of tumor cells from the site of origin. Thyroid tumor cells are destroyed during the metastasis cascade, releasing many tissue structural components into body fluids (Li et al., 2006). Metastasis can be determined by detecting markers of cancer cells in lymph nodes. Cytokeratin 19 (CK-19) is a low molecular weight cytokeratin that exists in a variety of tumors of epithelial and normal epithelial origin (Chen et al., 2018). Many studies have shown that CK-19 is highly expressed in differentiated thyroid carcinoma but is expressed at low or even no levels in benign thyroid tumors (Arcolia et al., 2017; Viana et al., 2020; Menz et al., 2021). Cyfra 21-1 is the proteolytic portion of CK-19. Elevated serum Cyfra 21-1 is an indicator of poor prognosis for many malignant neoplasms, including lung cancer, bladder cancer, and cervical cancer (Ma et al., 2015; Chen et al., 2018; Jin et al., 2019; Mei et al., 2019). Recent evidence suggests that the level of Cyfra 21-1 is associated with distant metastasis and tumor progression of thyroid cancer (Giovanella et al., 2008; Malapure et al., 2020). Therefore, detection of Cyfra 21-1 in lymph nodes may predict the metastasis of cancer cells to lymph nodes.

Lateral immunochromatography, also known as immunochromatographic strip (ICS) testing, uses classic immunochromatography analysis and reduces the time to obtain test results from hours to minutes. These tests require only simple equipment and do not require the use of advanced professional testing (Mitra and Sharma, 2021). Therefore, ICS tests are suitable for on-site testing. Since Beggs and Osikowicz first developed colloidal gold immunochromatography for the qualitative detection of human chorionic gonadotropin, the assay has been widely used in clinical trials (Beggs et al., 1990). However, there are currently no reports that indicate whether this method detects lymph node metastasis of malignant thyroid tumors. Therefore, the aim of this study was to develop a colloidal gold ICS test that can be used during surgery to detect and identify suspected lymph node metastasis from thyroid cancer.

## Materials and Methods

### Materials and Instruments

Anti-Cyfra 21-1 detecting antibodies, anti-Cyfra 21-1 capturing antibodies, and Cyfra 21-1 recombinant antigen were purchased from Shanghai LingChao Biological Co., Ltd. (Shanghai, China) and goat-anti-mouse IgG was purchased from ShengGong Biotech (Shanghai, China). Glass cellulose membranes (conjugate pad), polyester film (sample pad), absorption pads, and polyvinyl chloride (PVC) baseplates were also purchased from Shanghai JinBiao Biotechnology Co., Ltd. Nitrocellulose filter membranes were purchased from Life Sciences (California, United States). Chemical reagents, including gold chloride (HAuCl_4_·3H_2_O), sodium citrate (C_6_H_5_Na_3_O_7_·2H_2_O), sodium azide, ProClin 300, Tween 20, and polyvinylpyrrolidone K30 were purchased from Sigma (St. Louis, MO, United States). RIPA lysis buffer was obtained from Proteintech (Wuhan, China). A three-dimensional film cutting and gold spraying instrument, automatic cutting machine, shell pressing machine, and chromatography reader were purchased from Shanghai JinBiao Biotechnology Co., Ltd. (Shanghai, China).

### Preparation and Characterization of Colloidal Gold

Colloidal gold particles were prepared according to previously published methods (Frens, 1973). Briefly, under rapid magnetic stirring, 500 ml of ultrapure water was boiled, and then 2.5 ml of 0.5% trisodium citrate solution and 0.5 ml of 1 M sodium ascorbate were added simultaneously. After the reaction was allowed for 3 min, 50 µL of 100% chloroauric acid was added. The color immediately changed to that of wine red. After being boiled for 10 min, the colloidal gold solution was gradually cooled to room temperature and stored at 4°C for use in experiments. Colloidal gold particles were characterized by transmission electron microscopy (Talos L120C G2, Thermo Scientific, Netherlands), and the hydrodynamic diameter of colloidal gold was measured by nanoparticle size analyzer (Nano-S90, Malvern, Britain). The crystallographic characterization of nanoparticles was analyzed by a powder X-ray diffraction (XRD) spectrometer (Rigaku, SmartLab, Japan).

### Optimization and Preparation of Antibody–Colloidal Gold Conjugates

To determine the optimum conjugation pH, 1, 2, 3, 4, 5 and 6 μL K_2_CO_3_ (0.2 M) were added into six tubes containing 1 ml colloidal gold solution, and then the pH values were 5.9, 7.0, 7.8, 8.6, 9.6 and 9.9, respectively. After that, 2 μL detecting antibody solution (1.0 mg mL^−1^) was added into each tubes and mixed thoroughly for 30 min. The combination of colloidal gold and antibodies was detected using a Synergy H1 microplate reader (EnSpire). The optical density (OD) values were measured at 400–700 nm, and the optimum pH of the colloidal gold solution was obtained.

With adjusted optimum pH, 1, 2, 3, 4, 5 and 6 μg Cyfra 21-1 detecting antibodies were added into six tubes containing 1 ml colloidal gold solution, respectively. After the adequate agitation for 30 min, the OD values were measured, and the optimal concentration of the antibody was obtained.

Appropriate amount of Cyfra21-1 antibody was added to colloidal gold solution with optimum pH. After the mixture for 30 min, BSA (10%, 100 μL) was added to block the nonspecific conjugation. Then the mixture was centrifuged at 10,000 rpm for 20 min. The supernatant was carefully removed to discard the unconjugated antibody, and the pellet was re-suspended in 100 μL incubation buffer (0.01 M PBS) containing 1% BSA. Next, 1% polyethylene glycol-20000 (PEG-20000) was added to block the unreacted sites on the gold colloids, which was stored at 4°C for further experiments. Finally, the hydrodynamic diameter of colloidal gold was measured by nanoparticle size analyzer (Nano-S90, Malvern, Britain).

### Preparation of ICS

The colloidal gold ICS test comprised four components: a sample pad, a conjugate pad, a nitrocellulose membrane, and an absorbent pad. The sample pad (fiberglass membrane, Catalog No. RB65, JinBiao Biotech) was saturated with 0.01 M phosphate-buffered saline (PBS) solution containing 0.5% Triton X-100, 5% trehalose, 1% bovine serum albumin, 0.5% polyvinylpyrrolidone, and 0.01% Proclin 300 and then dried at 37°C for 16 h and stored in sealed storage containers at room temperature.

The conjugate pad (polyester film, catalog No. DL42, Shanghai JinBiao Biotech) was treated with a blocking solution of 0.01 M PBS containing 2% bovine serum albumin, 0.5% sucrose, 0.5% Tween 20, 0.5% polyvinylpyrrolidone K30, and 0.02% sodium azide (pH 7.4) and dried at 37°C before use. The colloidal gold probe was jetted onto the conjugate pad and dried at 37°C for 16 h and stored in a sealed storage container at room temperature.

Cyfra 21-1 capturing antibodies (2 mg mL^−1^, catalog No. L1C00705, Shanghai LinChao Biotech) and the goat anti-mouse antibody (0.5 mg mL^−1^, catalog No. D111121, ShengGong Biotech) were dispensed onto the nitrocellulose membrane (Shanghai JinBiao Biotech) in two discrete zones: one for the test line and the other for control line. An XYZ platform (Shanghai JinBiao Biotech) was used with a volume of 18 μL cm^−1^. The nitrocellulose membrane was dried at 37°C for 16 h and stored in dry containers at room temperature.

A PVC plate (catalog No. MT101B200429, Shanghai JinBiao Biotech) was used as the base of the ICS. The absorption pad, nitrocellulose membrane, conjugate pad, and sample pad were attached sequentially to the PVC plate with a 1–2 mm overlap. A cutting machine (Shanghai JinBiao Biotech) was used to cut the assembled plate into pieces 5 mm wide. Strips were stored in sealed storage containers at room temperature.

### Clinical Sample Collection and Processing

Patients with thyroid cancer treated in the Department of Otolaryngology Head and Neck Surgery at the First Affiliated Hospital of Anhui Medical University from May 2020 to May 2021 were included. The study was approved by the ethics committee of the First Affiliated Hospital of Anhui Medical University (5101128). All procedures were carried out in accordance with the principles of the declaration of Helsinki. All patients provided written informed consent. After excluding all other potential factors causing cervical lymph node enlargement, enlarged lymph nodes removed from patients with thyroid cancer during the operation were collected. On the basis of postoperative pathological findings, the samples were divided into an experimental group (lymph nodes with metastasis) and a control group (lymph nodes without metastasis). Every lymph node was separated into two parts, one part was sent to the pathology department for postoperative pathological assessment, and the other part was immediately placed in liquid nitrogen and then transferred to −80°C for later use in experiments.

### Clinical Sample Detection

Lymph node samples were cut into small pieces, and adipose and connective tissues were removed. An appropriate amount of pre-cooled PBS was added to a tube with the small pieces and centrifuged at 4°C and 700 g for 3 min. The resulting supernatant was discarded. The lymph node tissue was ground with an electric grinding pestle after 5 μL mg^−1^ of RIPA lysis buffer (PR20001, SanYing Biotech, Wuhan, China) was added, until no obvious tissue pieces were observed and the solution appeared homogeneous. The sample was then centrifuged at 4°C and 12,830 *g* for 3 min to precipitate tissue or cell fragments. The supernatant was retained as the total protein portion, and 60 µL of supernatant was added to each sample well for the ICS test. After 8–15 min, an image containing color signals appeared on the test paper. At 15 min, the ratio of color of the test line to that of control line (T/C value) was determined by tomography. If both the test and control lines were red, the sample was recorded as being positive. If the control line was red but the test line did not turn red, the sample was recorded as being negative. If the control line did not develop color, the ICS test was considered invalid.

### Immunohistochemical Analysis

Paraffin-embedded tissues were obtained, sectioned to a thickness of 3 μm, and subjected to IHC analysis. Immunohistochemistry was performed using the EnVision (DAKO; Hamburg, Germany) two-step system, diaminobenzidine color development, and hematoxylin counterstaining. The results were interpreted by at least two pathologists. The criteria for positive expression of Cyfra 21-1 were cells with brownish-yellow granules on the membrane and in the cytoplasm. Tissue sections with Cyfra 21-1–positive cells higher than 10% were considered positive, otherwise the tissue section was considered negative for the expression of Cyfra 21-1.

### Statistical Analysis

Data are expressed as means ± SEM, and all data were analyzed using GraphPad Prism software (version 7, San Diego, CA). The Probit model was applied to determine the detection limit of the ICS test, and simple linear regression was used to generate a standard curve of the recombinant protein level plotted against the T/C value. Statistical evaluation of the storage time of ICSs was performed using the unpaired Student’s *t*-test and analysis of variance. A two-sided value of *p* < 0.05 was considered statistically significant.

## Results

### Characterization of Colloidal Gold Particles

**FIGURE 1:**
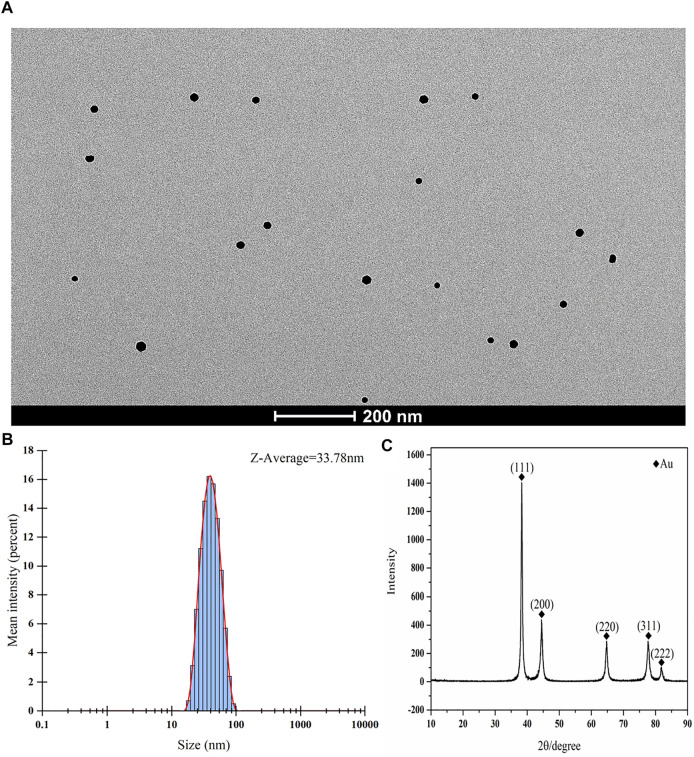
Characterization of colloidal gold. **(A–C) (A)** Representative TEM images of gold nanoparticle and **(B)** Size distribution of the colloidal gold particles showing an average hydrodynamic diameter of 33.78 nm. **(C)** XRD spectrum of the synthesized nanoparticles.

Chloroauric acid reduction was used to synthesize colloidal gold particles. During colloidal gold preparation, the solution changed from being colorless to appearing wine red, gold ions were reduced to gold atoms in solution, and the gold atoms immediately accumulated into colloidal gold. The resulting colloidal gold particles were evaluated by transmission electron microscopy and nanoparticle size analyzer. The colloidal gold particles were spherical and no colloidal gold particle agglomerations were found. The average hydrodynamic diameter of colloidal gold particles is 33.78 nm (Figures 1A,B), suggesting that the colloidal gold particles were stable in solution, thus satisfying the requirements of colloidal gold for use as probes. The crystal structures of colloidal gold particles were analyzed by the X-ray diffraction (XRD). The XRD pattern of Au showed the diffraction peaks at 2θ angles 38.187°, 44.385°, 64.576°, 77.566°, and 81.722° corresponding to the crystalline gold atomic planes (111), (200), (220), (311), and (222) (Figure 1C). XRD pattern did not contain impurity peak, which indicated the high purity of Au (JCPDS Card No. 99-0056).

### Conjugation Optimization and Size Distribution of Antibody-Gold Conjugates

**FIGURE 2:**
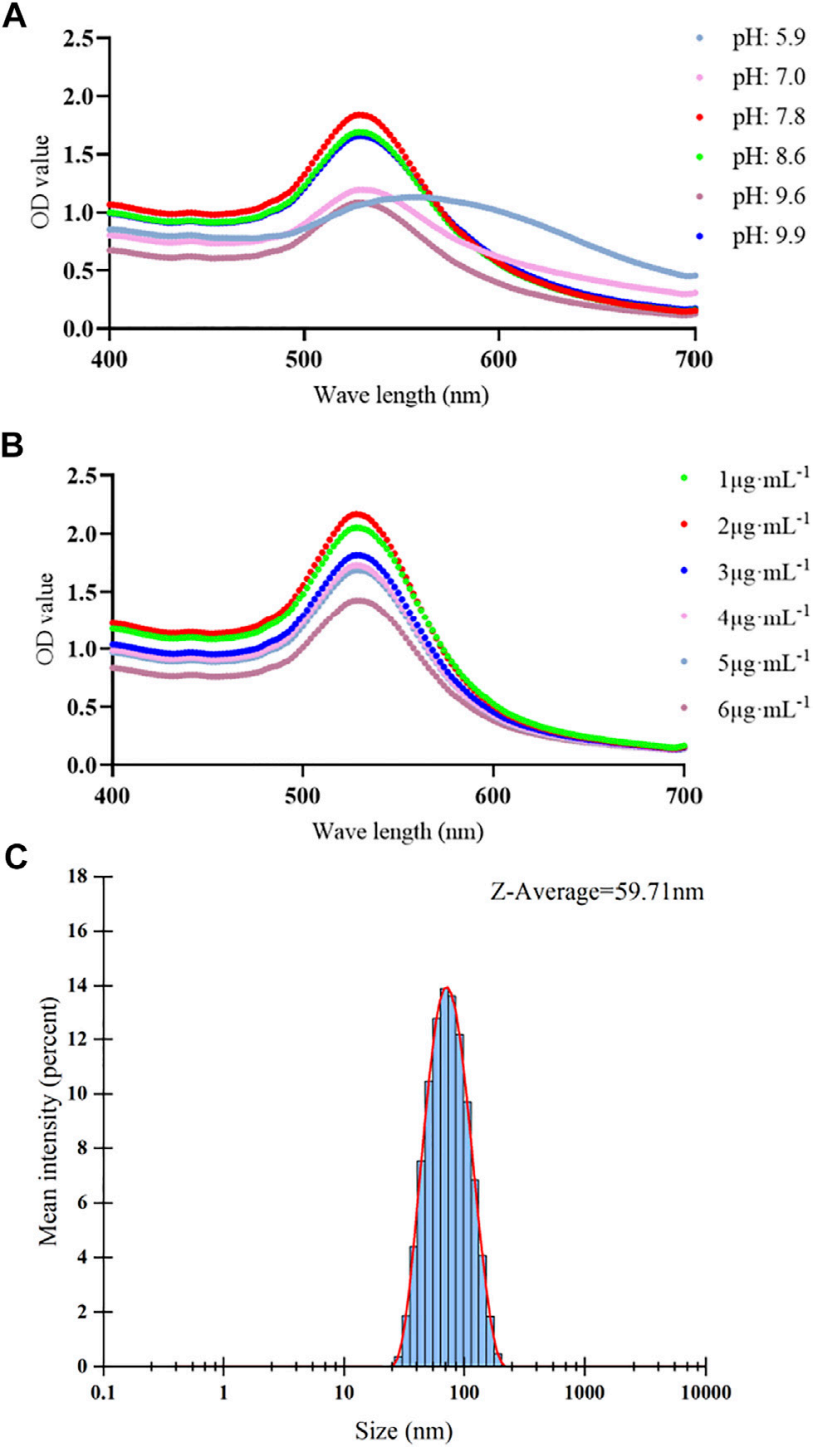
Optimization of pH value and antibody concentration and size distribution of antibody–colloidal gold conjugates. **(A–C) (A)** The different color lines showing the spectra absorption curves of different pH values. The maximum optical density (OD) value of spectra absorption curve was pH 7.8. **(B)** The different color lines showing the spectra absorption curves of different concentrations of Cyfra 21-1 detecting antibody. The maximum OD value of spectra absorption curve was 2 μg mL^−1^. **(C)** Size distribution of the antibody–colloidal gold conjugates showing an average hydrodynamic diameter of 59.71 nm.

The optimal pH value and concentration of Cyfra 21-1 detecting antibody for preparing antibody–colloidal gold conjugates were measured. After the optimization, the optimal pH was pH 7.8 (Figure 2A), and the optimal Cyfra 21-1 detecting antibody concentration was 2 μg mL^−1^ (Figure 2B). The colloidal gold conjugates were prepared under optimal conditions. The results showed that the average particle size of colloidal gold is 33.78 nm (Figure 1B). The average particle size of colloidal gold-antibody complex was 59.71 nm which is larger than the colloidal gold particle diameter indicating that the colloidal gold particle was successfully conjugated with the antibody (Figure 2C).

### Standard (Calibration) Curve for the ICS Test

**FIGURE 3:**
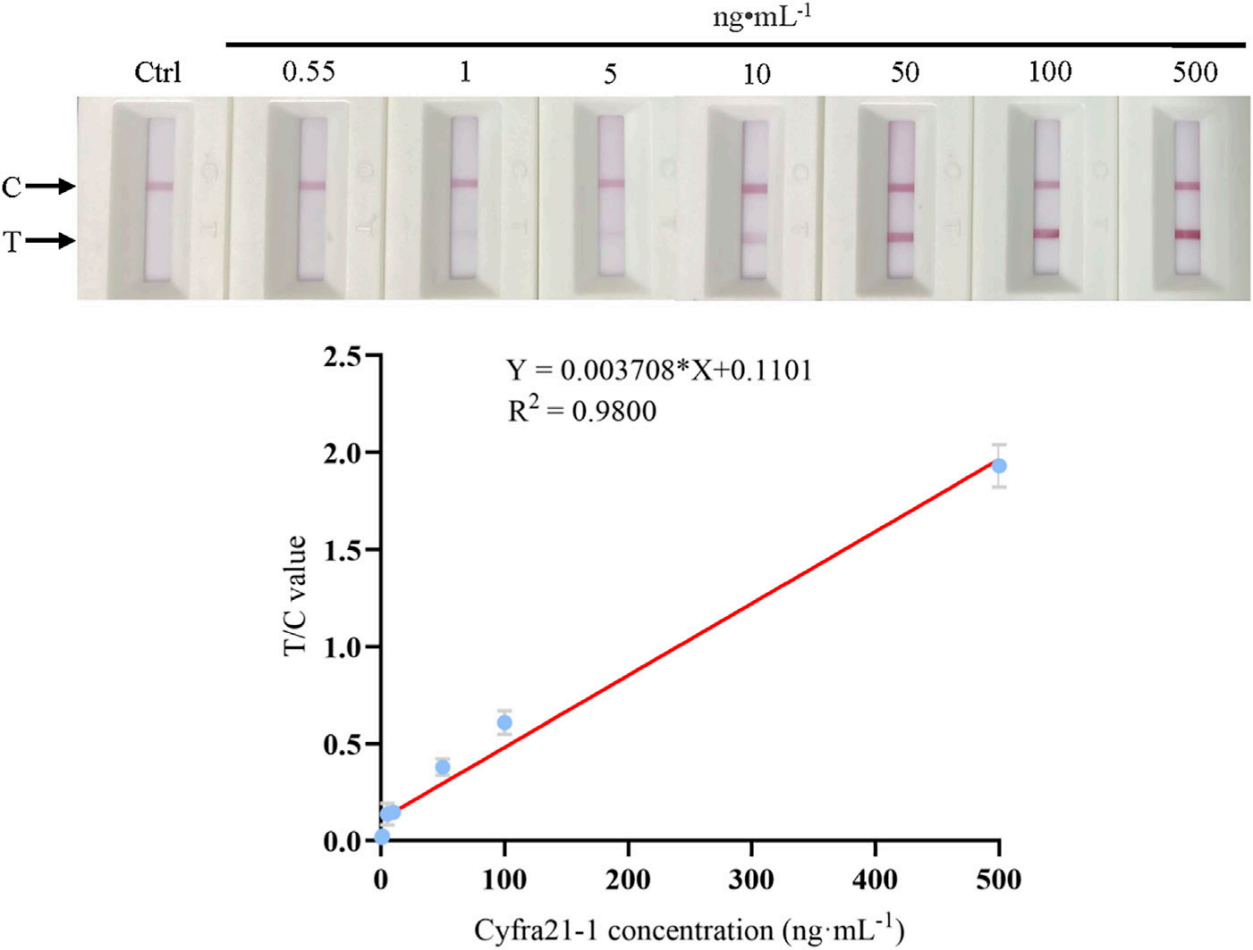
Standard (calibration) curve of the Cyfra 21-1 recombinant protein concentration and the T/C value of the immunochromatographic strip. The regression equation is Y = 0.003708 × X + 0.1101, with a correlation coefficient (*R* ^2^) of 0.9800. The minimum concentration visible to the naked eye on the immunochromatographic strip is 5 ng mL^−1^. From left to right, the Cyfra 21-1 recombinant protein concentration is 0.55–500 ng mL^−1^. C = control line, T = test line.

To obtain a concentration-response curve between the sample Cyfra 21-1 concentration and the T/C value, Cyfra 21-1 recombinant protein was diluted gradually, and 60 µL of each dilution was added to the ICS at room temperature. The values of the test and control lines corresponding to each concentration were detected after 15 min by using a fluorescence reader, and the T/C value was calculated. As shown in Figure 3, when the Cyfra 21-1 concentration was between 0.55 and 500 ng mL^−1^, there was a linear relationship between the concentration and T/C value (Y = 0.003708 × X + 0.1101; *R* ^2^ = 0.9800), which was used to determine the Cyfra 21-1 concentration of unknown samples. The minimum concentration visible to the naked eye on the ICS was 5 ng mL^−1^.

### Sensitivity of the ICS

To determine the sensitivity of the colloidal gold ICS, the concentration of Cyfra 21-1 recombinant protein was gradually diluted with tissue lysate to obtain 10, 8, 6, 5, 4, and 1 ng mL^−1^. RIPA buffer was used as the negative control. After mixing, 60 µL of Cyfra 21-1 recombinant protein was added to the ICS, which was left at room temperature for 15 min; the T/C value was determined, and the process was repeated 20 times. We used a Probit model to establish the detection limit of the ICS, which was 0.55–1.14 ng mL^−1^ (*p* = 0.996) (Table 1).

**TABLE 1:** Sensitivity of the immunochromatographic strip to Cyfra 21-1.

Concentration of Cyfra 21-1, ng·mL^−1^	No. of Positive Tests	No. of negative Tests	Total no. of Tests
0	0	20	20
1	0	20	20
4	1	19	20
5	3	17	20
6	8	12	20
8	19	1	20
10	20	0	20

Positive: The T/C value of the strip test was substituted into the standard (calibration) curve to determine the concentration of Cyfra 21-1 recombinant protein, and the result was the number of positive samples. Negative: The T/C value of the strip test was substituted into the standard (calibration) curve to determine the concentration of Cyfra 21-1 recombinant protein, and the result was the number of negative samples.

### Specificity of the ICS

**FIGURE 4:**
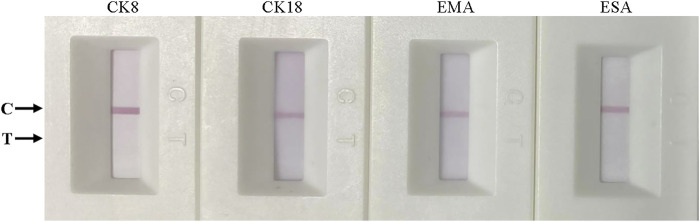
Specificity of the immunochromatographic strip. From left to right, cytokeratin 8 (CK8), cytokeratin 18 (CK18), epithelial membrane antigen (EMA), and epidermal surface antigen (ESA). All markers were tested at 500 ng mL^−1^.

To detect the specificity of the colloidal gold ICS, other marker proteins of epithelial cells, namely, cytokeratin 8, cytokeratin 18, epithelial membrane antigen, and epidermal surface antigen (Moll et al., 1982; Raphael et al., 1994; Balzar et al., 1999; Lau et al., 2004), diluted to a concentration to 500 ng mL^−1^ with RIPA buffer were assessed. After a 15-min reaction, only the control line appeared red; thus, there was no colloidal gold aggregation on the test line (Figure 4). These results indicated the specificity of ICS test for Cyfra 21-1 rather than for other markers of epithelial cells.

### Stability of the ICS

Colloidal gold ICSs were stored at room temperature for 6 months to evaluate their stability after storage. Cyfra 21-1 recombinant protein was detected at concentrations of 5, 50, and 500 ng mL^−1^, and the T/C values were recorded before storage and after 6 months of storage. Seven strips were tested for each concentration at each time point. As shown in Table 2, ICSs stored at room temperature for 6 months successfully detected Cyfra 21-1 at all three concentrations tested, with no loss of sensitivity.

**TABLE 2:** Stability of the immunochromatographic strip.

Storage Time (mo)	5 ng mL^−1^ Cyfra 21-1 (mean ± SD T/C)	50 ng mL^−1^ Cyfra 21-1 (mean ± SD T/C)	500 ng mL^−1^ Cyfra 21-1 (mean ± SD T/C)
0	0.082 ± 0.020	0.389 ± 0.050	1.811 ± 0.131
6	0.096 ± 0.028	0.406 ± 0.033	1.759 ± 0.074
*p* value	0.302	0.456	0.378

T/C indicates the value of the test line divided by the value of the control line. No significant differences were observed in the T/C values for tests conducted at the start of the experiment (0 months) and after 6 months of storage (*n* = 7).

### ICS and IHC Detection of Lymph Node Metastasis in Specimens

**FIGURE 5:**
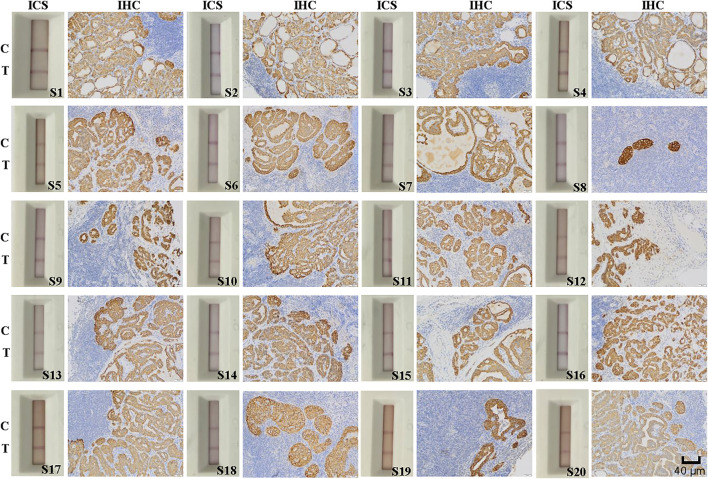
Immunohistochemical (IHC) staining and immunochromatographic strip (ICS) testing of lymph nodes deemed positive for thyroid cancer metastasis by using postoperative histopathology. Images show IHC staining for Cyfra 21-1 and ICS test results of 20 lymph nodes with tumor metastasis confirmed by postoperative histopathology. The sensitivity of the ICS was 100% compared with the histopathology results. C indicates the control line, and T indicates the test line. Specimen numbers are indicated as S1–S20.

**FIGURE 6:**
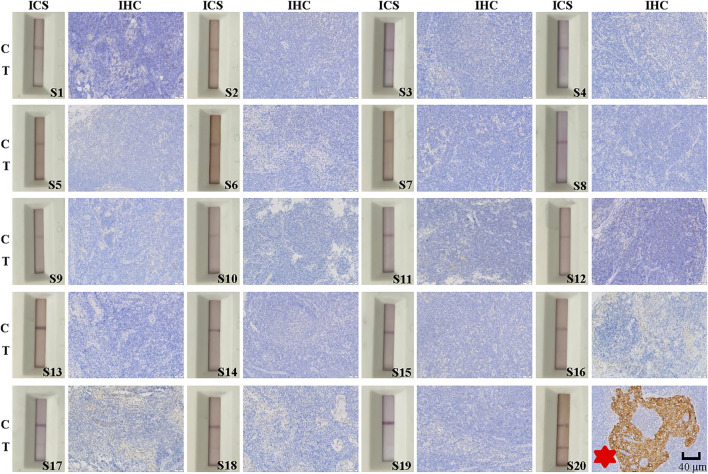
Immunohistochemical (IHC) and immunochromatographic strip (ICS) testing of lymph nodes without thyroid cancer metastasis as assessed by postoperative histopathology. Images show IHC staining for Cyfra 21-1 and ICS test results for 20 lymph node specimens without cancer metastasis as determined by postoperative histopathology. The sensitivity of the ICS test was 95% compared with histopathology results but 100% compared with IHC results. C indicates the control line, and T indicates the test line. Specimen numbers are indicated as S1–S20.

We obtained 20 lymph node specimens that were positive for cancer cell metastasis as determine by postoperative pathological findings and 20 specimens that were negative. We assessed the ability of the ICS test to accurately detect lymph node metastasis among the 40 specimens. For the 20 lymph node specimens deemed positive by postoperative pathology, the ICS test also indicated that all 20 specimens were positive for metastasis and 0 were negative (Figure 5). For 20 specimens deemed negative for metastasis by postoperative pathology, the ICS test indicated that 19 were negative and 1 was positive (Figure 6). Thus, the sensitivity of the ICS was 100% and the specificity was 95%.

Among the 40 lymph node specimens, 21 were determine to be positive for lymph node metastasis by IHC staining for Cyfra 21-1. Of them 20 specimens had been deemed positive for cancer cell lymph node metastasis by postoperative pathology, one had been deemed negative. In addition, 19 specimens were found to be negative for Cyfra 21-1 IHC staining, and all of these specimens and also been deemed to be negative for lymph node metastasis via postoperative pathology (Figures 5, 6). The same specimen (strip S20 in Figure 6) that had originally been deemed to be negative for lymph node metastasis by postoperative pathology was found to be positive in the present study by both Cyfra 21-1 IHC staining and our ICS test. Thus, the results of IHC staining for Cyfra 21-1 were 100% concordant with the results of the ICS test.

## Discussion

This study describes the development, testing, and validation of a colloidal gold chromatographic strip for simple, portable, and rapid detection of Cyfra 21-1 protein in lymph nodes. Comparison of the results of the strip with both postoperative pathological findings and IHC for Cyfra 21-1 results indicated that the developed ICS test showed excellent sensitivity and specificity. By reading the intensity of a colored line on the strip indicating an antigen and antibody response with a simple device, semi-quantification of the Cyfra 21-1 level in lymph node specimens could be obtained within 15 min, assisting in the determination of the need for lymph node dissection during thyroid cancer surgery.

During tumor metastasis, a large amount of Cyfra 21-1 is released into the extracellular fluid during the intermediate stage of epithelial cell apoptosis, resulting in increased Cyfra 21-1 levels in body fluids (Sheard et al., 2002; Li et al., 2006; Chen et al., 2018). At present, there are many methods to detect Cyfra 21-1 in blood, with a detection threshold as low as 9.08 fg mL^−1^ (Lu et al., 2021; Yang et al., 2021; Lv et al., 2022). Lei et al. developed an ICS test based on fluorescent microspheres for the rapid and quantitative detection of urinary Cyfra 21-1, which is in use for the diagnosis and prognostic monitoring of bladder cancer, confirming the reliable application of immunochromatographic technology in detecting Cyfra 21-1 levels (Lei et al., 2019). Recent studies have also shown that an increased Cyfra 21-1 level is associated with distant metastasis and tumor progression of thyroid cancer (Giovanella et al., 2017; Jeong et al., 2021). Determination of the Cyfra 21-1 level in the eluent from a fine-needle aspiration of the lymph node may improve the diagnosis of lymph node metastasis in patients with differentiated thyroid cancer (Lee et al., 2019). Therefore, detection of Cyfra 21-1 in lymph nodes of patients with thyroid cancer is a reliable way to determine metastasis of cancer cells.

In clinical practice, the classic method of confirming lymph node metastasis of thyroid cancer is postoperative histopathology. In the present study, the coincidence rate between the developed ICS test and postoperative pathology results was 100% for lymph nodes with cancer cell metastasis. However, the use of the ICS test reduced the number of false-negative results. A potential reason for the false-negative finding in the postoperative pathology results is that the spacing of histopathological sections is typically 2–3 mm; thus, some sections may show disease while others do not. In histopathological sections, only approximately 10%–12% of the entire lymph node tissue is assessed. By contrast, the ICS assesses the entire lymph node, that is, it is a comprehensive examination of all tissue in the lymph node, and can thus detect small lesions (Celebioglu et al., 2006). Many researchers have applied one-step nucleic acid amplification (OSNA) technology to detect levels of CK-19 mRNA in sentinel lymph nodes of patients with breast cancer to determine whether there is metastasis (Klingler et al., 2013; Babar et al., 2016; Shimazu et al., 2019). However, owing to the amplification of pseudogenes of genomic DNA using this technique, false-positive results are not uncommon. In addition, the detection time is approximately 50 min, and mRNA translation to protein is complex and may not match protein expression at any single point in time (Klingler et al., 2013; Huxley et al., 2015). Therefore, clinical application of OSNA is limited. Compared with OSNA technology, the use of our developed ICS decreased the detection time to 15 min or less, reducing overall operation time and thus cost and increased long-term benefits to patients. In addition, the cost of an ICS test is approximately $0.45.

This study was a preliminary study conducted in a single center with a relatively small sample size. Therefore, further large-scale studies are needed to verify the sensitivity and specificity of the developed ICS test.

## Conclusion

In this study, we developed a new ICS test using colloidal gold nanoparticles to semi-quantitatively detect the thyroid cancer biomarker Cyfra 21-1 in lymph nodes. Compared with traditional rapid detection technology, this ICS test has the advantages of high sensitivity, wider quantitative detection range, short detection time, and ease of use, making this ICS test faster and more convenient for surgeons to determine the need and scope of lymph node dissection during surgery for thyroid cancer.

## Data Availability

The raw data supporting the conclusion of this article will be made available by the authors, without undue reservation.
